# Navigating the intersection of 3D printing, software regulation and quality control for point-of-care manufacturing of personalized anatomical models

**DOI:** 10.1186/s41205-023-00175-x

**Published:** 2023-04-07

**Authors:** Naomi C. Paxton

**Affiliations:** Phil & Penny Knight Campus for Accelerating Scientific Impact, University of Oregon, Eugene, OR USA

## Abstract

3D printing technology has become increasingly popular in healthcare settings, with applications of 3D printed anatomical models ranging from diagnostics and surgical planning to patient education. However, as the use of 3D printed anatomical models becomes more widespread, there is a growing need for regulation and quality control to ensure their accuracy and safety. This literature review examines the current state of 3D printing in hospitals and FDA regulation process for software intended for use in producing 3D printed models and provides for the first time a comprehensive list of approved software platforms alongside the 3D printers that have been validated with each for producing 3D printed anatomical models. The process for verification and validation of these 3D printed products, as well as the potential for inaccuracy in these models, is discussed, including methods for testing accuracy, limits, and standards for accuracy testing. This article emphasizes the importance of regulation and quality control in the use of 3D printing technology in healthcare, the need for clear guidelines and standards for both the software and the printed products to ensure the safety and accuracy of 3D printed anatomical models, and the opportunity to expand the library of regulated 3D printers.

**Supplementary Information:**

The online version contains supplementary material available at 10.1186/s41205-023-00175-x.

## Background to 3D Printing anatomical models

3D printing, more accurately known as additive manufacturing, is playing an increasingly disruptive role in healthcare [1]. Broadly speaking, the fabrication technology uniquely lends itself to the clinical need to fabricate one-off products matching individual patient anatomy, and does not require high volumes to break-even as per traditional manufacturing [2]. 3D printing techniques rely on the additive deposition or fusion of material, layer-by-layer, to form 3D objects. This additive manufacturing paradigm unlocks tremendous design freedom and makes the technology ideally suited for fabricating patient-specific anatomic models or devices that typically entail complex geometries. 3D printing software often requires a CAD model as the input, which is ‘sliced’ into 2D layers and sequentially printed to form the 3D object [3].

**Fig. 1:**
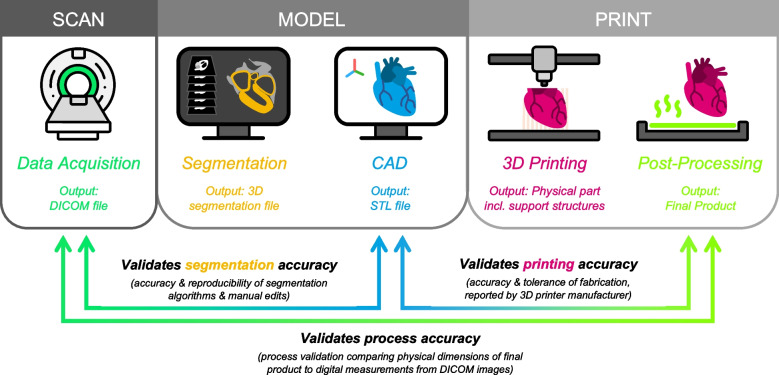
Overview of the process to design and fabricate 3D printed anatomical models, including acquisition of patient scan data in the form of DICOM, segmentation of the anatomy of interest, 3D modelling of the anatomy and CAD, 3D printing of a physical part and post-processing to clean, cure or remove support structures as necessary. Validation between specific outputs during the workflow is used to confirm the accuracy of specific processes

Over the last decade, 3D printing is being increasingly used for fabricating 3D models of patient anatomy, providing an added dimension to medical scan data visualization previously unachievable at the point-of-care using screen-based visualization technologies [4]. Advances in accessible 3D printing technology, in parallel to data handling and integrated storage systems, known in healthcare settings as ‘picture archiving and communication systems’ (PACS), are enabling hospitals and healthcare facilities to now rapidly translate imaging data out of the digital domain and into the physical domain (Fig. 1) [5]. To produce a 3D printed model from patient scan data, one must first obtain the scan data in a compatible format, such as a DICOM file, generated as the output viewing format from a variety of medical imaging techniques, such as computed tomography (CT) or magnetic resonance imaging (MRI) (Fig. 1, SCAN) [6]. Next, the scan data must be digitally segmented, which involves isolating and extracting the relevant anatomy from the rest of the scan data and background. This can be done by manually selecting the regions of interest on successive images, or through the use of automated algorithms or artificial intelligence (AI) driven tools that can extrapolated between multiple slices with a high degree of accuracy [7, 8]. Frequently, segmentation is performed using a combination of automated and manual tools (semi-automatic). Once the relevant anatomy has been isolated, it can be processed and converted into a format that can be used by a 3D printer, typically an STL or OBJ file (Fig. 1, MODEL). Finally, the 3D printer can be used to fabricate the physical model using a variety of materials, most typically plastics fabricated via stereolithography (SLA), fused filament fabrication (FFF) or binder jetting (BJ) due to low cost and accessibility in standard lab settings (Fig. 1, PRINT) [9].

3D printed models of regions of patient anatomy have many, often interchangeable names, such as “surgical planning models”, “anatomic models”, “medical models” or, common to regulatory information, “physical replicas of 3D models” referring to the *physical* production of models from *digital* 3D models generated using 3D modelling software [3, 10–12]. In this article, “3D printed anatomical models” has been adopted as a general and universally inclusive term for these models, regardless of application or intended use.

Due to their use in healthcare, with the opportunity to inform patient diagnosis, management, or treatment as diagnostic tool, these 3D printed anatomical models are of interest to regulatory bodies such as the US Food and Drug Administration (FDA). Currently, whilst 3D printed anatomical models prepared at the point-of-care are not currently considered medical devices themselves, the FDA has required that any 3D printed anatomical models *marketed for diagnostic use,* meaning those advertised for sale for the purposes of being used by a healthcare professional to diagnose a condition, should be prepared using software that has received FDA clearance [11]. Therefore, only a limited number of software platforms exist that have suitable clearance for the generation of anatomical models that can be produced in combination with validated 3D printers. Whilst the intended use of the software to produce physical replicas for diagnostic use is contained within a software’s 510(k) clearance documentation, there is no consolidated reporting mechanism for the specific combination of 3D printers and materials that have been validated using that software and details are sparsely reported by individual software or 3D printer manufacturers. Further, this list of cleared printers and materials in combination with the segmentation software is often developed for specific clinical indications and/or anatomic regions. This information is vital to healthcare professionals seeking to adopt 3D printing into surgical planning workflows and expand the accessibility of 3D printed anatomical models to improve patient care.

The current absence of a consolidated list containing information on cleared software and validated 3D printer combinations impairs accessibility and understanding of the landscape of 3D printing workflows suitable for clinical use. Therefore, the aim of this review article is to comprehensively survey software platforms that have been cleared by the FDA for the production of 3D printed anatomical models, alongside the range of 3D printers that have been validated for use to produce 3D printed anatomical models for diagnostic use. Additionally, this review aims to examine the suitability of current verification and validation methodology for the generation of such models, as well as to explore the potential for expanding the range of 3D printers that are validated for use with approved software.

## Medical device regulation for 3D modelling software

### US Software regulation for radiological software

Like many software platforms used in healthcare, 3D modelling software used to translate patient scan data into 3D models suitable for 3D printing is regulated by the FDA if they are intended to be used for diagnostic or therapeutic purposes [13]. Given the similarities in functionality to generic radiographic software, both types of software are used to create visual representations of medical data that can be used for diagnostic or therapeutic purposes, and as such, they have the potential to significantly impact patient health and treatment. In terms of their risk profile, radiographic software, as well as those with 3D printing-specific outputs, are generally classified as moderate risk (Class II) medical devices with in the ‘LLZ’ classification product code, depending on their intended use and the potential for harm if they do not function correctly. This process typically involves submitting a premarket notification, also known as a 510(k), to the FDA, which includes data demonstrating the safety and effectiveness of the software compared to an existing product on the market, known as a ‘predicate’. The FDA reviews this data and determines whether the software meets the necessary standards and can be cleared for sale. Alternatively, if a product has new features for which there is no predicate device already on the market, other application pathways may be required, such as de novo applications. The requirement for new software platforms to be subjected to some form of regulatory oversight is important because the use of 3D printed anatomical models produced from digital 3D models generated using these software platforms can have significant consequences for patient health and treatment if used for diagnosis or surgical decision making, and it is important to ensure that they are produced reliably and accurately.

### FDA-Cleared software for producing 3D printed models

Currently, there are seven software platforms on the market that have FDA clearance for producing 3D printed anatomical models. Table 1 summarizes these software platforms, with reference to FDA clearance documentation provided in Reference column. Each of these software include the generation of 3D printed anatomical models within their ‘intended use’ in combination with specific 3D printer brands, listed in column 3. 3D printed anatomical models produced using five of the software platforms have been cleared for diagnostic use “in conjunction with other diagnostic tools and expert clinical judgement” [14] for a range of clinical applications, namely orthopaedics (also referred to as musculoskeletal), craniomaxillofacial (incl. craniofacial and maxillofacial), and cardiovascular areas. However, AVIEW Modeler (Coreline Software Company) and Simpleware ScanIP (Synopsis) may only be used for “visualization and educational purposes” and do not currently possess clearance for diagnostic use. This means the models cannot be used by a healthcare professional to diagnose a patients’ condition based on the 3D printed model, however they may still be used for other activities within a healthcare setting such as surgical training and patient education [15, 16].

**Table 1:** List of 3D modelling with the intended use of producing 3D printed anatomical models, cleared by the FDA (Class II 510(k) pathway)

**Company**	**Software**	**Validated with Specific 3D Printers**	**Intended for Diagnostic Use**	**Intended Use Applications**						**Validation Accuracy **	**Reference**
				**Orthopaedic**	**(Cranio) Maxillofacial**	**Cardiovascular**	**Gastrointestinal**	**Genitourinary**	**Neurological**		
**Materialise**	Mimics Medical Mimics InPrint Mimics Enlight	Formlabs FORM 3/3B/3BL Stratasys J5 MEDIJET, J750/ J735/J850, OBJET30 PRIME HP MJF 580 Ultimaker S5	Yes	✓	✓	✓				< 0.2 mm	[14, 17]
**Synopsys**	Simpleware ScanIP Medical	Formlabs FORM 3B/3BL Stratasys J5 MEDIJET, J750/J850 HP MJF 580 Rize XRIZE	Yes	✓	✓	✓				Not reported	[18]
**3D Systems**	D2P	3D Systems ProJet CKP 660Pro 3D Systems ProJet 7000 HD 3D Systems ProJet MJP 5600 3D Systems ProX SLS 6100	Yes	✓	✓	✓	✓	✓	✓	Not reported	[19, 20]
**Ricoh**	Ricoh 3D Anatomic Models	Stratasys J5 MEDIJET, J750, F370	Yes	✓	✓					Not reported	[21, 22]
**Axial 3D**	Axial3D Cloud Segmentation Service	Formlabs FORM 3B Stratasys J5 MEDIJET, J750/J735 HP MJF 580, 540	Yes	✓	✓	✓				Not reported	[23, 24]
**Coreline Software Company**	AVIEW Modeler	Stratasys Objet260 Connex3	No							Not reported	[15]
**Medviso**	Segment 3DPrint	Formlabs FORM 3B+ Formlabs Fuse 1	Yes	✓	✓	✓				< 1 mm	[25, 26]

**Fig. 2:**
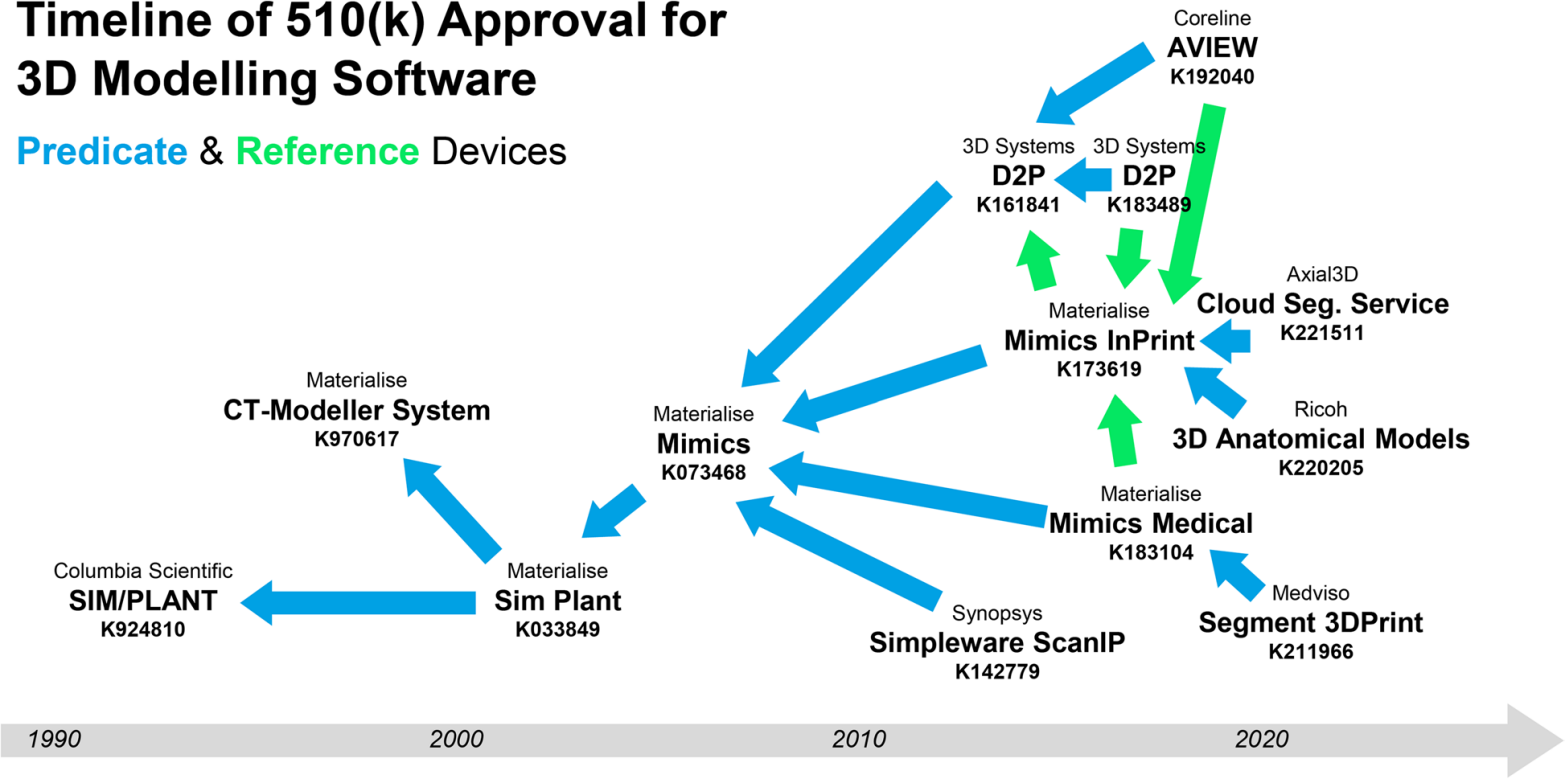
Timeline of 510(k) clearance for medical imaging software for producing 3D printed anatomical models. The company name, software name and 510(k) number are provided on a timeline, as well as arrows indicating a software application’s references to other software as a predicate or reference device in their 510(k) application

Materialise products (Mimics, Mimics InPrint and Mimics Medical) have played a critical role in establishing a benchmark for the safety and efficacy of these software platforms, with all other software platforms using a Materialise product as either a predicate or reference device for comparison of their safety and performance, and assessment of substantial equivalence (Fig. 2). Their 3D visualisation technology is underpinned by their platform 3D image viewing and surgical planning software developed in the 1990s for dental surgery applications. SIMPLANT remains in routine clinical use for dental surgery planning and surgical guide design after being acquired by a US dental equipment manufacturer, Dentsply Sirona [27].

The selection of validated 3D printers has largely been established through partnerships between software and 3D printing hardware manufacturers [21, 25], leading to a bespoke list of 3D printers being available for use in a validated and ‘on-label’ context. This list of 3D printers introduced in Table 1 has been expanded and reorganized in Table 2 to further explore trends in the growing selection, fabrication modalities and material availability. FormLabs and Stratasys are the most widely validated 3D printer brands, with their vat polymerization (VP) and material jetting (MJ) technology being marketed and applied widely for their capacity to produce accurate, flexible, multicoloured, or multi-component anatomical models [28–30]. Whilst the mean cost for one of the printers on the list is just under $100,000 USD ($98,612.50 USD, *n* = 16), several low-cost 3D printers are available, including the Ultimate S5 fused filament fabrication (FFF) system for use with PLA within the category of material extrusion (MEX) which, importantly, does not require any peripheral post-processing materials necessary for VP fabrication [30]. However, variation in the surface quality and material finish of each technique may render some techniques more suitable that others in addition to the accessibility of the price point. Intuitively, as 3D Systems is the only company to appear on both the list of software manufacturers and 3D printer manufacturers, they have exclusively validated their D2P software with several of their 3D printers [19]. Several printers on the list, including the FormLabs Fuse 1, HP580, 540, and, 3D Systems ProX SLS 6100, are capable of fabricating parts from nylon (PA11 or PA12) which is commonly used as a biocompatible material for tissue-interfacing applications such as surgical guides [31], however the regulatory complexities for producing such surgical tools extend beyond the scope of the aforementioned indications for use for anatomical models.

**Table 2:** List of validated 3D printers for the production of anatomical models using FDA-cleared software and estimates of their price points. (Binder Jetting (BJ), Material Extrusion (MEX), Material Jetting (MJ), Powder Bed Fusion (PBF), Vat Polymerization (VP))

Company	3D Printer	Type^a^	Approx. Printer Cost^b^ [USD]	Materialise Mimics Medical & InPrint	Simpleware ScanIP	D2P	Ricoh 3D Anatomical Models	Axial3D Cloud Segmentation Service	AVIEW Modeler	Segment 3DPrint
FormLabs	FORM 3	VP	$3.5 k	✓ (a)				✓ (b)		
	FORM 3B	VP	$4.3 k	✓ (a)	✓ (a)					
	FORM 3B +	VP	$3.8 k							✓ (q)
	FORM 3BL	VP	$13 k	✓ (a)	✓ (a)					
	FUSE 1	PBF	$18.5 k							✓ (p)
Stratasys	J5 MEDIJET	MJ	$60 k	✓ (c,e,g,h)	✓ (d,e,g,h)		✓ (q)	✓ (c,e,g,h)		
	J750	MJ	$250 k	✓ (d,f)	✓ (d,f,g,h)		✓ (q)	✓ (d,f)		
	J735	MJ	$200 k	✓ (d,f)				✓ (d,f)		
	J850	MJ	$200 k	✓ (d,f)	✓ (d,f,g,h)					
	OBJET30 PRIME	MJ	$36 k	✓ (g)						
	OBJET260 Connex3	MJ	$110 k						✓ (n)	
HP	MJF 580	PBF	$110 k	✓ (i)	✓ (i)			✓ (i)		
	MJF 540	PBF						✓ (i)		
Ultimaker	S5	MEX	$6 k	✓ (j)						
Rize	XRIZE	MJ + MEX	$55 k		✓ (k)					
3D Systems	ProJet CJP 660Pro	BJ	$60 k			✓ (l)				
	ProJet 7000 HD	VP	$100 k			✓ (m)				
	ProJet MJP 5600	MJ	$70 k			✓ (n)				
	ProX SLS 6100	PBF	$300 k			✓ (o)				
Validated Materials^c^				[17]	[16]	[19]	[11]	[23]	[15]	[32, 33]

^a^According to terminology defined in ISO/ASTM 52,900 Standard Terminology for Additive Manufacturing – General Principles – Terminology [34]

^b^Approximate starting price point based on public information [35]

^c^Each software platform has validated each printer with various combinations of materials. References are provided to the specific documentation to find the specific materials that have undergone validation testing

(a) FormLabs v4: Grey, Clear & White. (b) FormLabs v4: White, Clear; FL v2: Draft; FormLabs v1 Flexible. (c) VeroVivid™ Cyan, Magenta, Yellow; DraftWhite, VeroUltraClear™. (d) VeroBlackPlus, VeroClear, VeroCyan, VeroGrey, VeroMagenta, VeroPureWhite, VeroYellow. (e) Elastico™ Clear. (f) Agilus. (g) MED610. (h) MED615RGD. (i) Nylon 12: 3D HR CB PA 12. (j) PLA. (k) Rizium GF. (l) VisiJet® PXL™ + ColorBond™ infiltrant. (m) Accura® ClearVue™. (n) VisiJet CR-WT 200, VisiJet CE-NT. (o) DuraForm® ProX PA. (p) Nylon 11 Powder. (q) Not specified

In addition to the seven software platforms mentioned in Table 1, there are other programs that have similar capabilities for converting patient scan data into digital 3D models that can be used for 3D printing. However, these software platforms do not specifically describe the physical fabrication of models as an intended use of the software in their FDA clearance documentation (Table S1). These platforms include Advantage Workstation (AW) (GE Heathcare), that has been validated with Formlabs FORM 3B and 3BL printers [36], and Vitrea Advanced Visualization (Canon), validated with Stratasys Objet260 Connex3. IntelliSpace Portal 10 (Philips) and Synapse 3D (FUJIFILM) both market their software with 3D printing output capability [37, 38], whilst Dolphin 3D Surgery (Patterson Dental Supply), iNtuition (TeraRecon), Osirix MD (Pixmeo Sarl) and Syngo.via (Siemens) have demonstrated use for producing 3D printed anatomical models in the academic literature [39–43] (Table S1).

It is also necessary to distinguish between 3D printed anatomical models produced by a manufacturer for sale in the US, compared to those produced in-house by a hospital or other healthcare provider that are not marketed and sold. FDA regulation currently extends only to products produced for marketing and sale in the US and therefore, whilst it is best practice for hospitals producing 3D printed anatomical models to follow the FDA guidance requiring 3D printed anatomical models to be produced using cleared software, it is not presently a requirement. This nuanced guidance from the FDA is likely to undergo significant change over the coming years as the role of medical device manufacturer is clarified in the context of the growing trend and return towards point-of-care manufacturing [44]. Thought leaders in the 3D printing for medical application space strongly advocate for the use of approved software coupled with validated 3D printers in the interests of maintaining “very high standards” and minimizing risk to patient safety [45].

## 3D Printed product validation

### Inaccuracies in model design & fabrication

Reproducible dimensional accuracy is crucial for quality control of 3D printed anatomical models, particularly since they may be used to inform diagnosis and surgical decision-making that may impact patient safety and quality of care. Since these models are not considered medical devices, no harmonized quality control standards currently exist. Research teams and 3D printing facilities around the world have therefore developed and reported a variety of quality management methods, focusing on establishing reproducible dimensional accuracy of 3D printed parts. Dimensional accuracy is defined as the agreement between the measured and designed dimension of the 3D-printed part [46], and has vital clinical relevance for the quantitative use of these 3D models for characterising pathologies, such as tumours, aneurysms or other pathologies where dimensional fidelity strongly determines treatment pathway and prognosis. Therefore, each stage of the 3D printed anatomical model generation workflow (Fig. 1) requires careful analysis to determine the presence of controlled or uncontrolled sources of inaccuracy and therefore motivation for regulatory oversight.

**Fig. 3:**
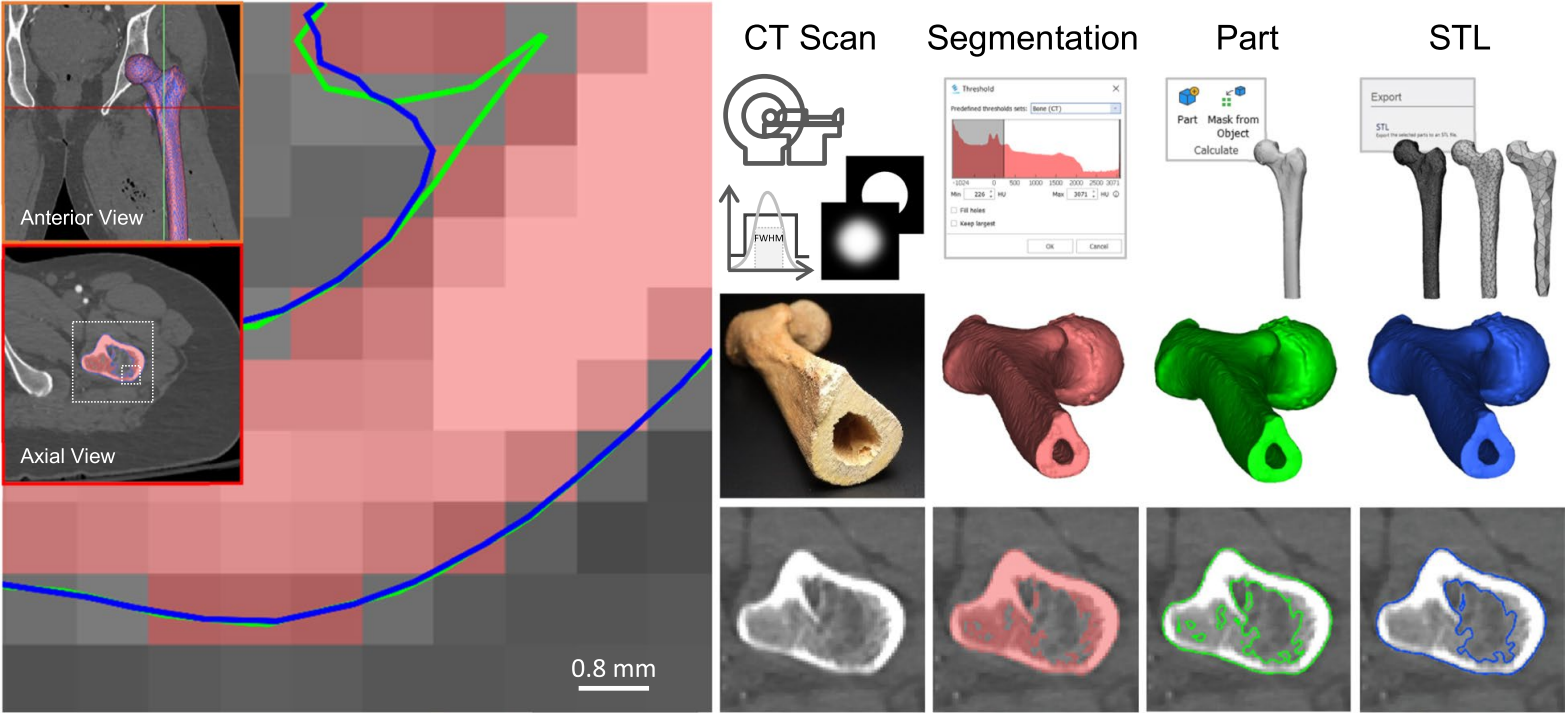
Comparison of 3D model morphology of a femur at high magnification. CT scan data (greyscale) was segmented (red) in Mimics (Materialise), converted to a ‘part (green) and exported to an STL file (blue) after ‘wrapping’ and floating body removal

Firstly, the image quality generated from CT and MRI scanning modalities is largely well-characterised, however the impact of imaging quality and parameters such as the choice of reconstruction kernel or slice reconstruction interval (SRI) have been shown to impact the mean absolute error between original models and 3D printed models [47]. Next, the digital process steps have the potential to introduce inaccuracy in the model design and interpretation of anatomical structures, particularly when performed by non-experts [48, 49]. Figure 3 demonstrates the source of estimation and inaccuracy between the original CT scan data of a femur versus the segmentation selection, ‘part’ and exported STL file. Whilst little difference is perceivable in the macroscopic views of the 3D models, at high magnification, the interpretation of the segmented pixel selection into a part and STL file yields a potential source of inaccuracy between the patient anatomy and produced model (Fig. 3). Several CAD tools are commonly used to prepare the part for final production, including the use of ‘wrap’ tools to close small holes in the 3D model, or mesh reduction to reduce and improve the quality of triangles comprising the STL model. These tools, in combination with the vast range of adaptation and manipulation tools available in CAD software such as 3-matic (Materialise) may impact the quality and accuracy of the 3D model compared to the patient anatomy and original scan data. This is consistent with previous reports demonstrating that different segmentation and part generation algorithms produce models with statistically significant variation in physical dimensions [50, 51]. This also further reinforces the accepted standard of practice for point-of-care 3D printing facilities to use software platforms cleared by the FDA in combination with validated 3D printers, since critical inaccuracies could step from several aspects of the workflow when using non-cleared and validated products, particularly when performed by non-radiologists, such as 3D printing technicians that do not have formal medical training.

**Fig. 4:**
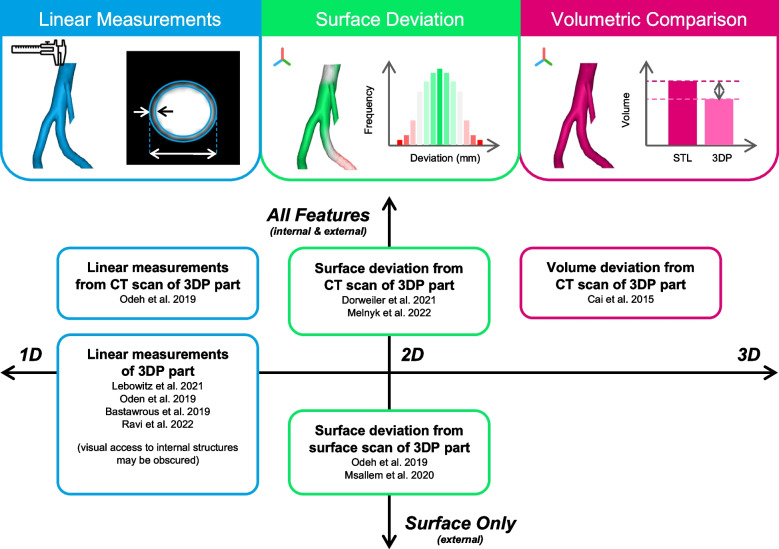
Summary of accuracy measurement techniques for validating the fabrication of 3D printed anatomical models. Linear measurements of anatomical features may be taken from a 3D scan of the 3D printed model or the physical model itself (blue) [42, 46, 52, 53], whilst optical or laser surface scanning allows 2D surface comparisons between anatomical features in the original scan data, STL file and physical model (green) [9, 42, 54, 55]. Finally, a ‘residual volume’ metric is proposed for 3D quantification of model accuracy (pink) [56]

Finally, dimensional accuracy of the final 3D printed models may be evaluated using a range of technologies, including callipers, photographic measurements, surface scanning, photogrammetry, coordinate measuring machines (CMMs), or CT scans, summarised in Fig. 4 [42]. Many studies evaluating accuracy focus on a single pathology or region of anatomy [9, 42, 47], and it has been highlighted that further research is needed to evaluate the accuracy of anatomic models across a more generalised range of anatomical regions [46].

### Validation & quality control methods

Whilst formalised quality control systems for 3D printing anatomical models in hospital have not yet been mandated by the FDA, several methodologies have been proposed in the academic literature, ranging from versatile guidance for routine manufacturing workflows, through to systematic academic studies reporting vital fundamental validation where the true anatomical accuracy has been directly measured from cadaveric samples [42, 52]. Since the true patient anatomy is rarely accessible during routine clinical cases, the DICOM scan data is widely accepted as the ground truth, to which the STL file and 3D printed part are compared (Fig. 1). Comparison of the DICOM file to STL file provides validation information on the accuracy of the segmentation and CAD processes, validating the software tools used to generate the digital 3D model. This validation is included in the validation and verification testing performed by FDA-cleared software platforms listed in Table 1 and validates the suitability of these platforms to accurately translate the 3D scan data into 3D models. At this stage, radiologist oversight is recommended to ensure the quality of the digital model [57]. Next, the STL file is 3D printed to generate the physical model, the accuracy of which compared to the STL file is intrinsic to the 3D printer itself, the material, the paired slicing software, printing mechanism, upkeep and maintenance, and may not be specific to the design being printed. This should be independently and routinely validated using standardized models using manufacturer-specific guidance [58]. Full process validation is therefore critical, ensuring that the final printed product is within an acceptable tolerance from the original DICOM data (Fig. 1).

Since the DICOM file (sliced 2D images), STL file (3D digital model) and final printed part (3D physical model) exist in different spatial as well as physical or digital domains, several metrics for comparison have been utilized: 1D linear measurements, 2D surface measurements, and 3D volumetric measurements (Fig. 4). Measurements on the final 3D printed part may be performed directly, in the case of linear measurements using callipers, or via re-visualization of the part using 3D surface scanning, such as optical, photogrammetry or laser scanning, or CT scanning, offering a continuum of spatial information at a variety of resolutions depending on the specific equipment used [59].

Industry leaders have widely supported the use of callipers to perform linear measurements directly on 3D printed outputs compared to digital linear measurements performed on the DICOM and STL files for routine quality control [46, 60]. These measurements are routinely performed on macroscopic dimensions of large components or wall thicknesses of hollow or tubular structures. These measurements may be compared to the STL file or original DICOM dataset, as shown in Fig. 1, with a tolerance of < 1 mm deviation between physical model and original data widely considered to be acceptable in the literature for diagnostic models [9, 46, 61]. However, such measurements on specific anatomical features of personalized models cannot be readily compared between cases. Therefore, the inclusion of standardized ‘landing blocks’ of a specific dimension added into the 3D model has been proposed by Ravi et al. (2022) to enable reproducible and comparable measurements between models of varying geometry and clinical application [46]. The tolerance threshold is much higher for devices such as anatomic guides that have to fit on the target bony anatomy compared to anatomic models used for diagnostic purposes. Other more comprehensive techniques such as surface and volume measurements based on scans of the physical part play a vital role in process establishment, enabling comparison from digital scan data of the printed product compared to segmentation and STL data. These techniques are comprehensive and enable accuracy characterisation of the accuracy of all features of the part, notably thin internal features that may be inaccessible for physical measurement. However, their role in routine quality control may be limited due to cost and time inefficiency compared to physical measurements with callipers [56].

## Conclusion & future directions

### 3D Printed anatomical models driving hospital-based manufacturing

As technology and the technological competency of healthcare providers for producing 3D printed anatomical models continue to advance, it is likely that FDA guidance will evolve to reflect these changes. The FDA may consider several dynamic factors when updating its guidance in the coming years, including the development of new applications, validation techniques, feedback from key stakeholders, such as surgeons, 3D printing experts and patient groups, as well as changes in the international regulatory landscape. This is particularly pertinent given the proximity of the technologies underpinning 3D printed anatomical model manufacturing to those capable of producing other personalised medical devices and equipment that fall under medical device manufacturing regulation.

The growing demand for personalised medical devices such as surgical implants has strongly driven the requirement for point-of-care manufacturing, both to minimize lead times for manufacturing personalized devices, as well as cybersecurity concerns to reduce data-sharing with third parties outside of the healthcare providers’ systems in the process of designing and manufacturing personalized devices. These new challenges intrinsic to the technological capability offered by 3D printing for producing personalized devices are stimulating a growing conversation within regulatory bodies to reconsider how healthcare providers can also act as medical device manufacturers.

### Availability of 3D printers

Beyond regulatory considerations, the availability of 3D printers that have been validated for use in conjunction with cleared 3D modelling software remains limited, as demonstrated in Tables 1 and  2. Only a small subset of the available types of 3D printing techniques are represented in the list of validated printers, as well as an even smaller cohort of the thousands of brands and models of 3D printers on the market capable of producing 3D printed anatomical models are validated and marketed for use in producing anatomical models. Strategic partnerships between software providers and 3D printer manufacturers have motivated the validation of specific printers with software platforms [16, 17, 62], however in the absence of validation testing methods used by these providers and manufacturers in the public domain, the list of available printers may remain limited. The prevalence of expensive (> $100,000 USD) printing equipment, with disproportionately few low cost options with respect to the range available on the market is a limiting factor for the acceleration of 3D printing facility establishment in hospitals, despite low-cost models having similar clinical relevance than those produced on high-cost equipment [63–65].

### Reimbursement & economics

Finally, a parallel challenge to accelerating the adoption of 3D printed anatomical models, in addition to regulatory and technological considerations, is the economical proposal. This has recently been the topic of an excellent editorial by Prof Frank Rybicki (University of Cincinnati) who examines the intersection of regulation and reimbursement in the current landscape of hospital-based manufacturing [57]. In July 2019, the American Medical Association (AMA) defined four new Current Procedural Terminology (CPT®) codes relating to 3D printed anatomical models and surgical tools. CPT® codes are a “uniform language for coding medical services and procedures to streamline reporting” [66], and the inclusion of specific codes relating to 3D printed models and guides presents and exciting step forward towards routine adoption and use of 3D printed models in healthcare settings. Specifically relating to 3D printed anatomical models, “codes 0559 T and 0560 T represent reimbursement for the production of individually prepared 3D printed models that can be made from one or more components and unique colors and materials” and can be used to bill for the production of these products during patient care [66]. However, the codes are currently ‘temporary’ Category III codes and therefore health insurers are not obliged to reimburse for these codes, nor is a specific value assigned to the code for reimbursement. It is therefore at the discretion of individual health insurers whether they choose to reimburse for 3D printed anatomical models and if so, for how much. A survey of the over 300 US health insurers’ [67] reimbursement schedules suggests that only 15 health insurers currently choose to reimburse for these specific CPT® codes, to an average value of $91.78 US per model (*n* = 15) [68–70]. The Veterans Health Administration reimburses the highest amount of the surveyed insurers, to a maximum of $372.78 US [71]. Coupled with their nationally leading network of on-site 3D printing facilities [72], including as a compliant medical device manufacturer [73], this poses an insightful estimate into the feasible cost of routinely produced 3D printed anatomical models based on the ability for the VHA to produce and bill for these models in-house. However, the comprehensive costs associated with producing anatomic models maybe substantially higher as demonstrated in a recent study where the average cost of producing anatomic models across 11 clinical indications at the point-of-care was $2180 and $2467 when outsourced to industry [74].

Ultimately, further research, validation testing methods and regulatory oversight will accelerate the availability of validated and cleared workflows for producing personalized surgical planning models for point-of-care manufacturing, propelling 3D printed anatomical models into routine clinical use. This article has sought to provide a consolidated summary of FDA-cleared software platforms specifically suited towards the generation of 3D printed anatomical models, as well as the 3D printing models currently validated for use with the FDA-cleared software. The sources of inaccuracy contributing to the risk profile of using non-cleared software and hardware combinations are also discussed, finally summarizing the currently accepted techniques for validating the entire scan-to-print pathway, alongside specific aspects of the manufacturing process to produce 3D printed anatomical models. This resource therefore seeks to enable further adoption of safe and effective point-of-case 3D printing for surgical planning models and expand their application towards routine adoption in healthcare settings globally.

### Supplementary Information

**Additional file 1:** **Figure S1.** Decision tree for inclusion criteria for 3D modelling and segmentation software into the consolidated list (Table 1) or supplementary list (Table S1). **Table S1.** FDA-cleared 3D modelling and segmentation software with the capability to generate STL files suitable for 3D printing, however 3D printed models as outputs not listed as ‘intended use’ in FDA documentation.

## Data Availability

All data generated or analysed during this study are included in this published article and its supplementary information files.
