# Correction: Navigating the intersection of 3D printing, software regulation and quality control for point-of-care manufacturing of personalized anatomical models

**DOI:** 10.1186/s41205-023-00194-8

**Published:** 2023-11-17

**Authors:** Naomi C. Paxton

**Affiliations:** Phil & Penny Knight Campus for Accelerating Scientific Impact, University of Oregon, Eugene, OR USA

**Correction to: 3D Printing in Medicine (2023) 9:9**

10.1186/s41205-023-00175-x

Following publication of the original article [1], errors were identified in Table 1. The spelling of the company name Synopsys has been corrected. In addition, software from this company with FDA clearance for 3 applications at the time of the initial publication has been corrected. The spelling of the Ricoh software name has been corrected, and their validated 3D printers have been corrected to add Stratasys F370. The Materialise software Mimics Enlight was added.

A corrected Table 1 is provided.

The original article [1] has been corrected.

**Table 1:** List of 3D modelling with the intended use of producing 3D printed anatomical models, cleared by the FDA (Class II 510(k) pathway)

Company	Software	Validated with Specific 3D Printers	Intended for Diagnostic Use	Intended Use Applications						Validation Accuracy	Reference
				Orthopaedic	(Cranio) Maxillofacial	Cardiovascular	Gastrointestinal	Genitourinary	Neurological		
**Materialise**	Mimics Medical Mimics InPrint Mimics Enlight	Formlabs FORM 3/3B/3BL Stratasys J5 MEDIJET, J750/ J735/J850, OBJET30 PRIME HP MJF 580 Ultimaker S5	Yes	✓	✓	✓				< 0.2 mm	[14, 17]
**Synopsys**	Simpleware ScanIP Medical	Formlabs FORM 3B/3BL Stratasys J5 MEDIJET, J750/J850 HP MJF 580 Rize XRIZE	Yes	✓	✓	✓				Not reported	[18]
**3D Systems**	D2P	3D Systems ProJet CKP 660Pro 3D Systems ProJet 7000 HD 3D Systems ProJet MJP 5600 3D Systems ProX SLS 6100	Yes	✓	✓	✓	✓	✓	✓	Not reported	[19, 20]
**Ricoh**	Ricoh 3D Anatomic Models	Stratasys J5 MEDIJET, J750, F370	Yes	✓	✓					Not reported	[21, 22]
**Axial 3D**	Axial3D Cloud Segmentation Service	Formlabs FORM 3B Stratasys J5 MEDIJET, J750/J735 HP MJF 580, 540	Yes	✓	✓	✓				Not reported	[23, 24]
**Coreline Software Company**	AVIEW Modeler	Stratasys Objet260 Connex3	No							Not reported	[15]
**Medviso**	Segment 3DPrint	Formlabs FORM 3B+ Formlabs Fuse 1	Yes	✓	✓	✓				< 1 mm	[25, 26]
