# Supplementary material for: Navigating the intersection of 3D printing, software regulation and quality control for point-of-care manufacturing of personalized anatomical models
Source: 3D Print Med. 2023 Apr 7;9:9. doi: 10.1186/s41205-023-00175-x (PMC10080800; doi:10.1186/s41205-023-00175-x)
Supplement: Supplementary file 1 — Additional file 1: Figure S1. Decision tree for inclusion criteria for 3D modelling and segmentation software into the consolidated list (Table 1) or supplementary list (Table S1). Table S1. FDA-cleared 3D modelling and segmentation software with the capability to generate STL files suitable for 3D printing, however 3D printed models as outputs not listed as ‘intended use’ in FDA documentation. [file 41205_2023_175_MOESM1_ESM.docx]

**SUPPLEMENTARY INFORMATION**

**Navigating the intersection of 3D printing, software regulation and quality control for point-of-care manufacturing of personalized anatomical models**

Naomi C Paxton^1*^

^1^ Phil & Penny Knight Campus for Accelerating Scientific Impact, University of Oregon, Eugene OR, USA

* Correspondence: [npaxton@uoregon.edu](mailto:npaxton@uoregon.edu)

**Does the software have functionality to translate patient scan data into an STL file (via segmentation)?**

**YES**

**NO**

**Out of scope for this review**

**Is the software FDA cleared?**

510(k) or de novo
Class IIa, Produce Code: LLZ

**Out of scope for this review**

**Is 3D printing a physical replica of an anatomical model listed as an ‘intended use’ of the software?**

**Software listed in Supp Table**FDA-cleared software not intended
for 3D printing as an output.

**YES**

**NO**

**NO**

**Software listed in Table 1**

**YES**

**Are the 3D printed model outputs cleared for diagnostic use?**

**YES**

**What are the intended applications?**

**Software listed in Table 1,**alongside indication for diagnostic use and intended applications, as described in 510(k) clearance documentation.

**NO**

**Software listed in Table 1,**with note stipulating limitation on
use for diagnosis

**What 3D printers have been validated for use with this software?**

Validated 3D printed are listed in
Tables 1 and 2.

***Figure S1.*** *Decision tree for inclusion criteria for 3D modelling and segmentation software into the consolidated list (****Table 1****) or supplementary list (****Table S1****).*

***Table S1.*** *FDA-cleared 3D modelling and segmentation software with the capability to generate STL files suitable for 3D printing, however 3D printed models as outputs not listed as ‘intended use’ in FDA documentation.*

| **Company** | **Software** | **Validated with Specific 3D Printers** | **Reference** |
| --- | --- | --- | --- |
| Anatomage Inc. | Anatomage | N/A | [1] |
| Canon | Vitrea® Advanced Visualization | Stratasys Objet260 Connex3 | [2] |
| Fovia Inc. | F.A.S.T. RapidPrint | N/A | [3] |
| Fujifilm Corporation | Synapse 3D | N/A | [4] |
| GE Healthcare | Advantage Workstation (AW) | Formlabs FORM 3B/3BL | [5] |
| itk-SNAP | itk-SNAP | N/A | [6] |
| Patterson Dental Supply Inc. | Dolphin 3D Surgery™ | N/A | [7] |
| Philips | IntelliSpace Portal 10 | N/A | [8] |
| Pixmeo Sarl | Osirix MD | N/A | [9] |
| Siemens | Syngo.via | N/A | [10] |
| TeraRecon | iNtuition | N/A | [11] |
| Thermo Fisher Scientific | Amira | N/A | [12] |

**Supplementary References**

1. Anatomage (2023) Virtual Dissection Table - 3D Anatomy Platform - Anatomage Table. https://anatomage.com/.
2. Canon (2023) Product Information – 3D Printing. https://www.vitalimages.com/3d-printing/.
3. Fovia (2023) F.A.S.T. RapidPrint. https://fovia.com/rapidprint/.
4. FUJIFILM Medical Systems USA (2021) Synapse 3D Product Data Sheet
5. Formlabs (2019) Formlabs and GE Healthcare Collaborate to Offer Customer End-to-End Solution for 3D Printed Patient Models. https://formlabs.com/company/press/formlabs-ge-healthcare-collaborate-offer-end-to-end-solution-for-patient-models/.
6. ITK-SNAP (2018) ITK-SNAP. http://www.itksnap.org/pmwiki/pmwiki.php.
7. Michelinakis G (2017) The use of cone beam computed tomography and three dimensional printing technology in the restoration of a maxillectomy patient using a dental implant retained obturator. J Indian Prosthodont Soc 17:406. https://doi.org/10.4103/JIPS.JIPS_106_17
8. Philips (2018) IntelliSpace Portal 10 Clinical Datasheet
9. Buffinton CM, Baish JW, Ebenstein DM (2022) An Introductory Module in Medical Image Segmentation for BME Students. Biomed Eng Educ 2022 1–15. https://doi.org/10.1007/S43683-022-00085-0
10. Mandolini M, Brunzini A, Facco G, et al (2022) Comparison of Three 3D Segmentation Software Tools for Hip Surgical Planning. Sensors (Basel) 22:. https://doi.org/10.3390/S22145242
11. Christensen A, Wake N (2019) Wohlers Report 2019: Medical Image Processing Software
12. Life Science Research | Thermo Fisher Scientific - US (2023) Amira Software . https://www.thermofisher.com/us/en/home/electron-microscopy/products/software-em-3d-vis/amira-software.html.
